# ADAM9 is present at endothelial cell - cell junctions and regulates monocyte – endothelial transmigration

**DOI:** 10.1016/j.bbrc.2017.09.089

**Published:** 2017-11-18

**Authors:** William R. English, Richard J. Siviter, Martin Hansen, Gillian Murphy

**Affiliations:** a University of Cambridge Department of Oncology, Cancer Research UK Cambridge Institute, Li Ka Shing Centre, Robinson Way, Cambridge, CB2 0RE, UK; b Tumour Microcirculation Group, Department of Oncology and Metabolism, The Medical School, University of Sheffield, Beech Hill Road, Sheffield S10 2RX, UK

**Keywords:** ADAM9, Endothelial, Permeability, VE-Cadherin, Monocyte, Transmigration, ADAM, A Disintegrin And Metalloproteinase, EGFP, Enhanced Green Fluorescent Protein, HA, hemagglutinin, ELISA, Enzyme Linked Immunosorbent Assay, VE-Cadherin, Vascular Endothelial Cadherin/Cadherin 5, ZO-1, zona occludens −1, Fc, Fc region of IgG

## Abstract

We have found that A Disintegrin And Metalloproteinase-9 (ADAM9) localises to cell-cell junctions with VE-Cadherin in confluent endothelial monolayers. Co-cultures of cells separately transfected with ADAM9-EGFP or ADAM9-HA showed expression is required in two adjacent cells for localisation to cell-cell junctions suggesting the ADAM9 ectodomain may self-associate. A direct interaction between ADAM9 ectodomains was confirmed using recombinant proteins and an ELISA based method. As the ADAM9 ectodomain can also exist as a soluble form physiologically, we examined if this could inhibit endothelial functions dependent on cell-cell junctions. The soluble ADAM9 ectodomain could not increase endothelial monolayer permeability or inhibit monocyte-endothelial adhesion, but could inhibit monocyte-endothelial transmigration. These novel findings point to ADAM9 playing an important role in endothelial cell biology that is distinct from the other ADAMs.

## Highlights

• ADAM9 is a component of cell-cell junctions.
• ADAM9 must be expressed by both adjacent cells for cell junction localisation.
• ADAM9 can self-associate via its ectodomain.
• The soluble ADAM9 ectodomain inhibits monocyte-endothelial transmigration.

## 1 Introduction

The A Disintegrin And Metalloproteinase (ADAM) family of proteins consists of 33 members that regulate an array of cellular processes including cell-cell fusion, cell adhesion and migration [1]. The proteolytically active members regulate these and other cellular processes through ectodomain shedding of cell surface cytokines, growth factors and receptors [1], [2]. ADAMs have also been shown to be integral to the regulation of cell-cell contact dependent signalling, adhesion and migration via a variety of mechanisms. This may be via proteolysis of cell-cell junction proteins including N-cadherin, E-Cadherin, VE-Cadherin and Ephrins [3], [4] or via their association with integrins [5]. These interactions are also important regulators of vascular endothelial cell function. ADAMs have been shown to localise to cell-cell junctions, including in endothelial cells [6], [7], [8]. ADAM10, 15, 17, and 28 have been shown to regulate leukocyte transmigration across endothelial monolayers [9], [10], a process that is dependent on proteolysis, regulation of phosphorylation of adherens junction proteins or interaction with integrins. In some instances, ADAMs have been found to be secreted as soluble forms, including ADAM9 and ADAM15 that can also influence cell adhesion and migration [11], [12], [13]. We have previously demonstrated that ADAM9 is able to shed angiotensin-converting enzyme from endothelial cells stimulated with lipopolysaccharide via a TNF-TNFR dependent mechanism [14]. However, the localisation of ADAM9 in endothelial cells is not characterised and it is not known if it can regulate other aspects of endothelial biology e.g. endothelial monolayer permeability, leukocyte-endothelial adhesion or transmigration. In this article we investigated the localisation of ADAM9 in human umbilical vein endothelial cells (HUVEC) to further understand its role in the regulation of endothelial cell biology, leading to the identification of a new function for ADAM9 in endothelial monolayers.

## 2 Materials and methods

### 2.1 Cell culture

Pooled donor HUVEC were purchased cryo-preserved at passage 2 from TCS Cellworks and cultured in the supplied large vessel endothelial culture medium up to a maximum of 12 population doublings (approx. passage 4 to 5). 293T cells were obtained from the Cancer Research UK collection and cultured in DMEM, 10% FCS v/v, 500 μg ml^−1^ l-glutamine and 100 units ml^−1^ penicillin and 100 μg ml^−1^ streptomycin. THP-1 were purchased from ECACC and cultured in RPMI, 10% foetal calf serum (FCS) v/v and 100 units ml^−1^ penicillin and 100 μg ml^−1^ streptomycin. All cells were cultured at 37 °C, 5% CO_2_, 100% humidity.

### 2.2 Immunofluorescence staining of cell monolayers

HUVEC were cultured on glass coverslips at the times indicated and fixed with methanol at −20 °C for 5 min prior to labelling in phosphate buffered saline (PBS) with 10 μg ml^−1^ of antibodies to ADAM9 (Triple Point Biologics, RP3-ADAM9, USA) and either VE-Cadherin (clone 75/Cadherin 5), ZO-1 (Clone 1) or γ-Catenin (Clone 15) purchased from BD Transduction Laboratories. Cells were incubated with the primary antibody for 1 h before washing 3 times with PBS and then incubated with either anti-rabbit Alexa488 or anti-mouse Alexa546 conjugated secondary antibodies (1:1000, Invitrogen, UK) for 1 h, washed and mounted in Mowiol. Images were obtained using a Zeiss LSM 510 confocal laser-scanning microscope.

### 2.3 Cloning of ADAM9 chimeras and mutants

Mouse ADAM9 cDNA, ADAM9-Fc ectodomain (ECTO-Fc) in pEE12 were obtained as described previously [14]. The ECTO-Fc was cloned from pEE12 into pCDNA3.1 zeo+ using PCR. The soluble ADAM9 ectomain (ECTO, residues 1 to 698), was cloned from pEE12 using PCR into the vector pSecTag2B allowing the addition of a myc-[His]_6_ tag, producing ECTO-His. Alternatively, mouse ADAM9 ECTO-His was purchased from R&D Systems. To generate ADAM9-EGFP and ADAM9-HA, the complete ADAM9 cDNA was cloned from pEE12 by PCR removing the stop codon and then cloned in frame into pEGFP-N2 and pCDNA3.1 zeo + respectively, thus generating C-terminal tagged proteins. All PCR steps were verified by sequencing.

### 2.4 Transient transfection of 239T cells with cDNAs expressing ADAM9 chimeras

293T cells were chosen as we had previously found they express very low levels of ADAM9 [14]. 293T cells were seeded at 3 × 10^5^ in six well tissue culture plates at the same time as the cDNA-Fugene6 (Roche GmbH) transfection mixture. The cells were separately transfected with either ADAM9-HA or ADAM9-GFP cDNAs. 24 h after transfection, the cells were washed 3 times with fresh medium and then cultured for a further 24 h. The separately transfected cells were then trypsinised and re-seeded together at a 1:1 ratio on 10 mm cover glasses placed in 12 well tissue culture plates at 5 × 10^4^ cells per well, resulting in the formation of a confluent monolayer. After 24 h, the cells were fixed in 4% w/v paraformaldehyde in PBS for 10 min, washed with PBS before incubating in 100 mM glycine, 0.01% Triton X-100 (Thermofisher, UK) for 5 min. Cells were then incubated with a rabbit anti-HA polyclonal antibody (5 μg ml^−1^, F-7, Santa Cruz) for 1 h, washed 3 x in PBS before incubation with anti-mouse Texas red secondary antibody (1:500, Jackson Immunochemicals) for 1 h in PBS and a further 3 washes in PBS before mounting in Mowiol.

### 2.5 Purification of soluble ADAM9 and mutants

The various soluble forms of ADAM9 were transiently transfected into 293T cells by calcium phosphate precipitation. ECTO-Fc was purified from cell culture medium by protein A affinity chromatography at 4 °C. Cell culture medium was sterile filtered, adjusted to 1.65 M NaCl, 0.9 M Glycine, pH 8.8 and loaded onto a protein A column pre-equilibrated with 1.65 M NaCl, 0.9 M glycine pH 8.8 and washed with the same buffer. ECTO-Fc was eluted with 0.1 M glycine, 0.5 M NaCl pH 2.5 and immediately neutralised with Tris-HCl before dialysis against 50 mM Tris-HCl, 50 mM NaCl, pH 7.4. Myc-[His]_6_-tagged ADAM9 ectodomain (ECTO-His) was purified by NiNTA affinity chromatography. Medium was loaded overnight at 4 °C on a NiNTA-agarose column pre-equilibrated with medium. The column was washed with 50 mM Tris-HCl, 500 mM NaCl, 10 mM Imidazole, pH 8.0 and eluted with 50 mM Tris-HCl, 500 mM NaCl, 200 mM Imidazole, pH 8.0. Protein concentration was estimated by BCA assay (Thermofisher) and protein purity by reducing SDS-PAGE followed by coomassie blue staining. Alternatively protein was detected using western blotting detected using enhanced chemiluminescence (ECL). The anti-Fc antibody was from Jackson Immunochemicals (1:1000) and the anti-myc antibody was from Cell Signalling Technology (1:1000, 9B11). Protein was then stored in aliquots at −80 °C. The mouse ADAM9 ectodomain with a C-terminal [His]_6_ tag was also purchased from R&D systems.

### 2.6 ADAM9-ADAM9 ELISA

ADAM9 ectodomains were coated at 2 μg ml^−1^ in 0.1 M Na_2_CO_3_ pH 9.8 overnight at 4 °C onto NUNC-immuno maxisorb 96-well plates. Plates were washed 3 x in PBS-Tween 20 (0.05%). Unspecific binding was blocked by 1 h incubation with 200 μl PBS, 1% Tween-20 v/v, 1% BSA w/v, 1% w/v fat free milk powder. This buffer is also used for all incubations. ADAM9 with a different tag was then incubated for 1 h at RT before washing 3 times with PBS-Tween 20. Bound ADAM9 was detected by incubation for 1 h at RT with the appropriate antibody at 5 μg ml^−1^ and washed 3 times. Finally detection was achieved with incubation for 1 h at RT with the appropriate HRP-conjugated secondary antibody. The plate was then washed 6 times with PBS-Tween 20. The plate was developed by incubation with 100 μl TMB ONE ready-to-use substrate (KEM EN TEC) and the reaction stopped by addition of 100 μl 0.2 M sulphuric acid. Absorbance was measured at 405 nm with a TECAN Spectrafluor Plus microtitre plate reader.

### 2.7 Endothelial permeability and monocyte-endothelial transmigration assay

100 μl 5 μg ml^−1^ Fibronectin (Sigma Aldrich) in PBS was used to coat 6 mm diameter transwells with a 3 μm pore size (Corning Costar) for 1 h. 1 × 10^5^ HUVEC were then seeded in large vessel endothelial cell medium (LVECM) and cultured for 4 days to allow a confluent monolayer to form. For permeability assays, HUVEC were incubated with ECTO-His at 3 μM for 2 h or with 300 μM H_2_O_2_ for 30 min [15]. 45 kDa FITC-dextran was added to the upper well at 0.8 mg ml^−1^ for 30 min [16]. Fluorescence of the medium in the lower well was measured in 96 well plates using a TECAN Spectrafluor Plus microtitre plate reader with excitation/emission wavelengths of 435/495 nm. For transmigration assays, THP-1 cells were resuspended in serum free medium in the presence of 2 μM CMFDK green tracker dye (Invitrogen) for 30 min at 37 °C before centrifuging at 300 x *g* and resuspension in LVECM at 2 × 10^6^ cell ml^−1^. 2 × 10^5^ THP-1 cells were added to the transwell insert in 100 μl and migration was allowed to proceed for 4 h at 37 °C. ECTO-Fc or Fc alone were pre-incubated with the HUVEC monolayer and THP-1 cells for 30 min at 3 μM prior to addition of the THP-1 cells to the monolayer. After 4 h the transwell insert was removed and the medium from the lower chamber centrifuged at 300 x g. The medium was removed and the pellet lysed in 100 μl cell lysis buffer. This was combined with a 100 μl lysis buffer wash from the bottom of the insert and lower chamber. Fluorescence of cell lysates was measured on a TECAN Spectrafluor plus fluorescence plate reader in 96 well microtitre plates with excitation/emission wavelengths of 435/495 nm.

### 2.8 Monocyte – endothelial adhesion assay

The monocyte adhesion assay was adapted from methodology described by McGinn et al. [10]. Briefly, HUVEC were grown to confluence in 96 well tissue culture plates. THP-1 cells were labelled with CMFDK green tracker dye as described above. HUVEC were pre-incubated with 3 μM ECTO-Fc for 30 min before addition of 1 × 10^5^ THP-1 cells, ensuring the final concentration of ADAM9 remained 3 μM by adding it to the THP-1 cells. THP-1 were incubated on the HUVEC monolayers for 30 min before removal of medium containing non-adherent cells and fixing with 4% w/v paraformaldehyde for 20 min. The wells were washed with PBS before fluorescence was measured on a TECAN Spectraflor Plus with excitation/emission wavelengths of 435/495 nm.

### 2.9 Statistical analysis of data

Grouped data was analysed using one-way ANOVA with a Tukey multiple comparisons post-test. Pair-wise comparisons were made using an unpaired *t*-test with Welch's correction. Analysis was performed using GraphPad Prism version 7.0c for Mac OSX, GraphPad Software, La Jolla California USA, www.graphpad.com. *, ** and *** are used in the figures to indicate P < 0.05, 0.001 and 0.0001 respectively.

## 3 Results

### 3.1 ADAM9 localises to cell-cell junctions and co-localises with cell - cell junction proteins in endothelial cells

**Fig. 1:**
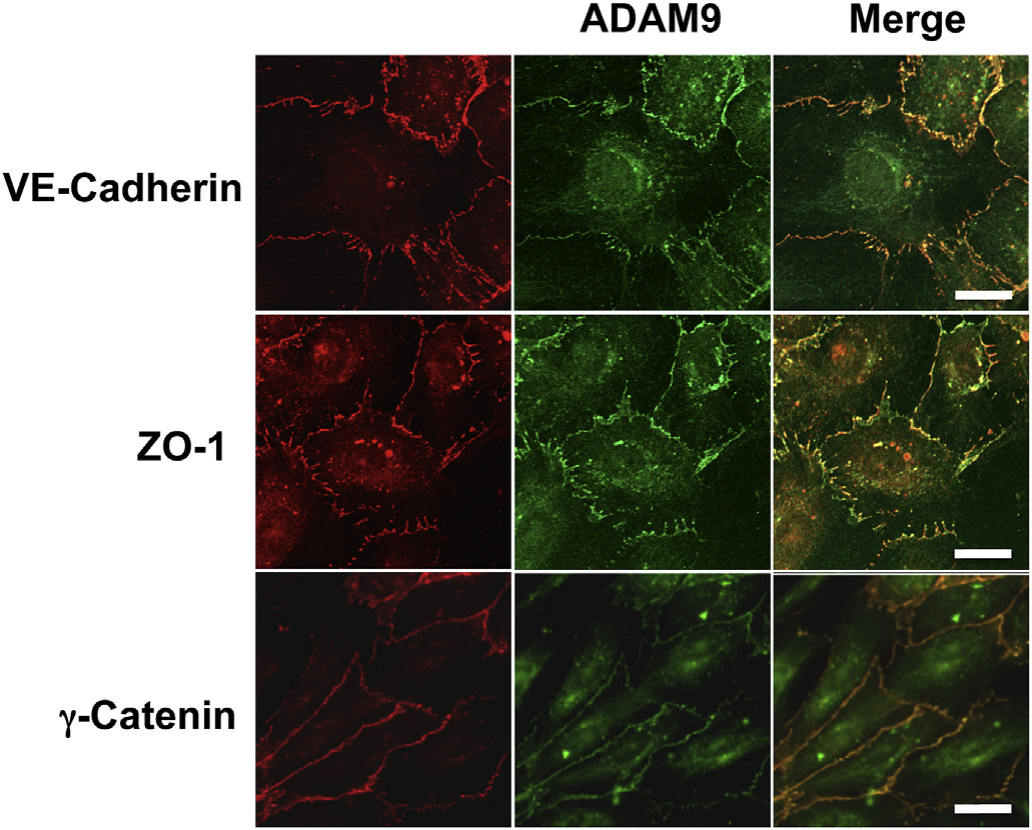
*ADAM9 localises to cell-cell junctions in HUVEC.* Confocal images of confluent HUVEC stained for ADAM9 (green) and either VE-Cadherin, ZO-1 or γ-Catenin (red). Merged image shows overlap in staining in yellow. Scale bars are 20 μm. (For interpretation of the references to colour in this figure legend, the reader is referred to the web version of this article.)

**Fig. 2:**
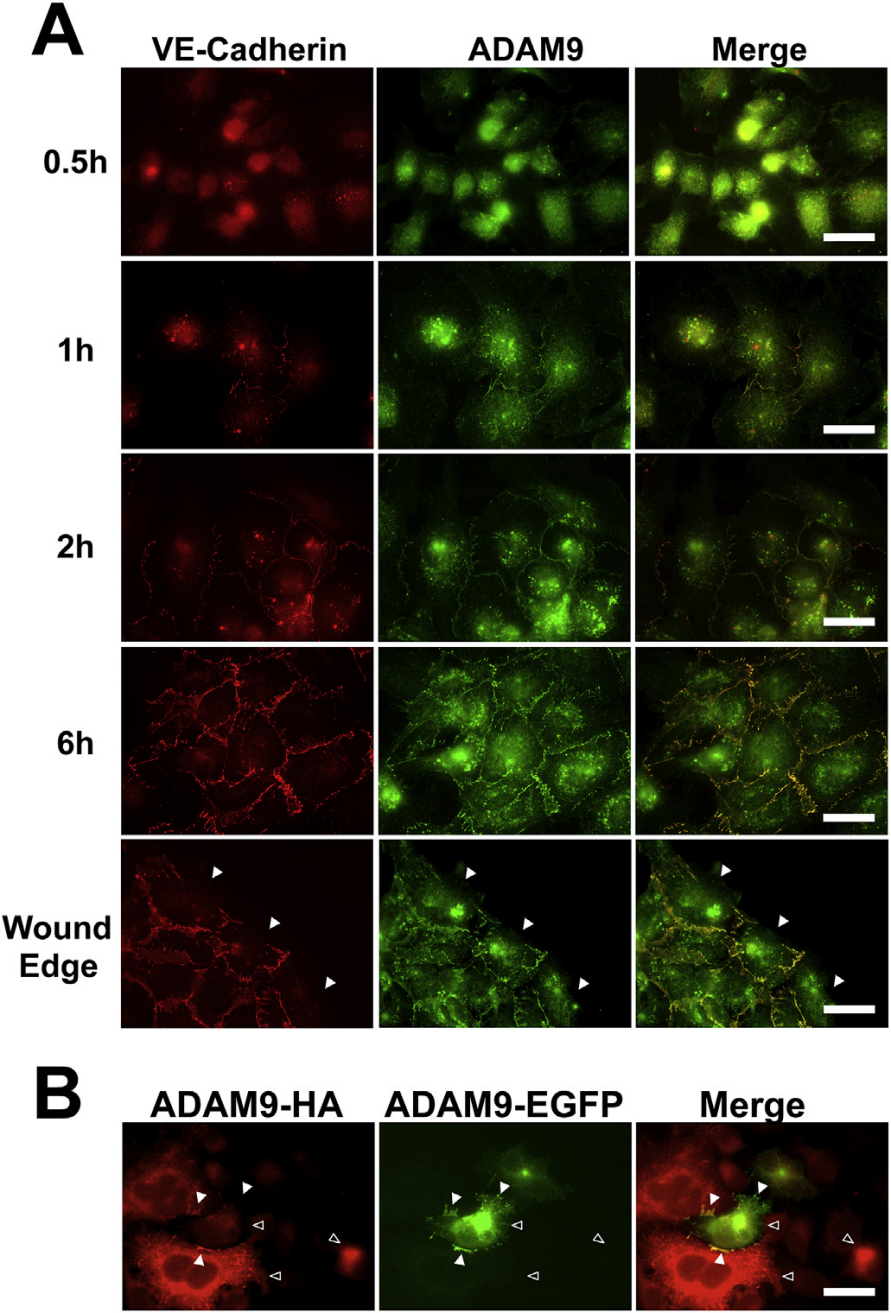
*ADAM9 moves from a cytosolic localisation to cell-cell junctions in HUVEC during formation of a confluent monolayer and must be expressed by two adjacent cells.***A.** HUVEC were seeded at high density to ensure formation of a confluent monolayer after cell spreading was complete and fixed at 0.5–6 h intervals before staining for VE-Cadherin (red) and ADAM9 (green) and imaged using confocal microscopy. In addition, a confluent monolayer was wounded by forming a scratch through the monolayer and stained after 30 min for VE-Cadherin and ADAM9. The white arrows indicate areas where both ADAM9 and VE-Cadherin staining is lost at the wound edge in regions were no adjacent cell is present. Images in the right hand column are the merged images showing overlap in staining between VE-Cadherin and ADAM9 in yellow. Scale bars are 45 μm. **B.** 293T cells were transfected separately with either ADAM9-HA (red) or ADAM9-EGFP (green) before trypsinisation, mixing and culturing as a confluent monolayer as described in the materials and methods section. Cells stained with anti-HA (red). Solid arrows indicate localisation of ADAM9 at cell-cell contacts between adjacent cells expressing ADAM9-HA and ADAM-EGFP. Open arrows highlight the lack of ADAM9 staining at cell-cell junctions where one adjacent cell is not transfected. Scale bar is 45 μm. (For interpretation of the references to colour in this figure legend, the reader is referred to the web version of this article.)

ADAM9 was detected in confluent HUVEC monolayers by immunofluorescence staining at cell-cell junctions, as well as within vesicles throughout the cytosol. Co-staining with cell junction proteins VE-cadherin, γ-catenin and ZO-1 confirmed that ADAM9 localises to cell-cell junctions (Fig. 1). Similar to ADAM15 [6], ADAM9 staining at cell junctions appeared concomitantly over time with VE-Cadherin when HUVEC were seeded onto glass at confluency, and was absent when cell-cell contact was lost after introducing a scratch wound in confluent monolayers (Fig. 2A).

### 3.2 ADAM9 must be expressed by adjacent cells to localise to cell - cell junctions

We next investigated if ADAM9 localisation at cell-cell junctions was dependent on ADAM9 being expressed by two adjacent cells. ADAM9 chimeric cDNAs were constructed with either EGFP or HA tags at the C-terminus. 293T cells were transiently transfected separately with either ADAM9-EGFP or ADAM9-HA before trypsinisation and co-culturing together. ADAM9 was only seen at cell-cell junctions when cells were expressing tagged ADAM9, and only when both adjacent cells were expressing tagged ADAM9 as seen by co-localisation of GFP and HA tagged ADAM9 to cell-cell junctions (Fig. 2B).

### 3.3 ADAM9 can self-associate through ectodomain interactions

**Fig. 3:**
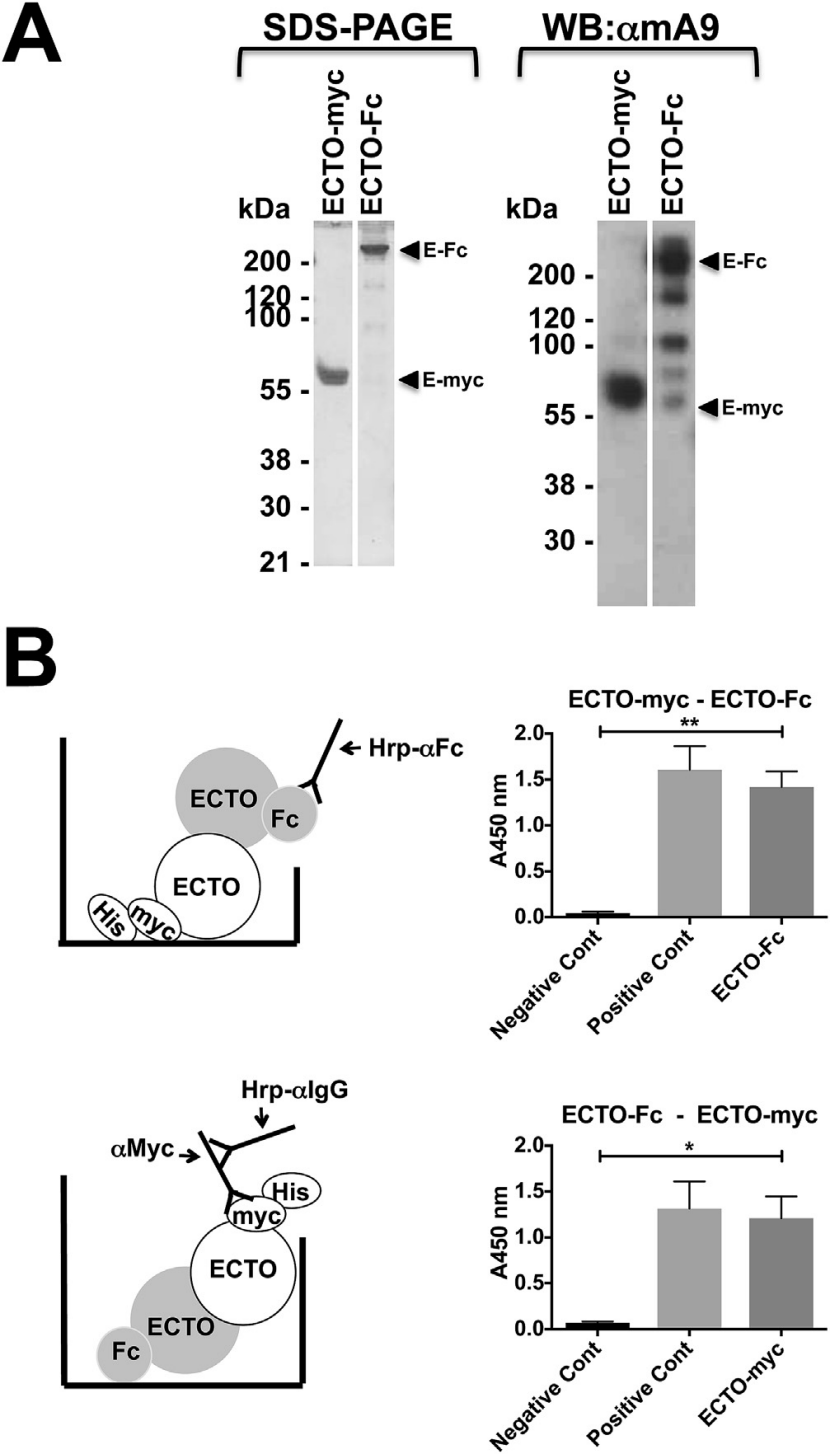
*ADAM9 can self-associate through ectodomain interactions.***A.** Purified soluble ADAM9 recombinant ectodomain (residues 1–698) with a c-terminal IgG Fc (ECTO-Fc) or myc-[His]_6_ (ECTO-myc) tags were run on reducing SDS-PAGE and stained with coomassie blue (left) or detected using Western blot and ECL with an anti-mouse ADAM9 antibody (right). **B.** Diagrams showing the tagged ectodomain used to coat the microtitre plate surface, the tagged ectodomain added in solution and the corresponding detection antibodies used, paired with the results obtained. The negative control in each case was the absorbance obtained in the absence of the ADAM9 ectodomain in the soluble phase and the positive control was detection of the ADAM9 ectodomain in the solid phase with the appropriate tag directed antibody. Data shown is absorbance (A450 nm, arbitrary units). Error bars are ±SEM. * = P < 0.05. ** = P < 0.01.

Our findings raised the possibility that ADAM9 may have the ability to self-associate through ectodomain interactions. To address this, recombinant ADAM9 ectodomains (ECTO) were expressed in 293T cells with myc-[His]_6_ (His) or IgG Fc (Fc) C-terminal fusions and purified from the cell conditioned medium (Fig. 3A). By coating 96 well microtitre plates with either ECTO-His or ECTO-Fc we found that ECTO-Fc or ECTO-His proteins, respectively, showed association with the ADAM9 ectodomain coated on the plate surface (Fig. 3B).

### 3.4 The soluble ADAM9 ectodomain inhibits endothelial - monocyte transmigration

**Fig. 4:**
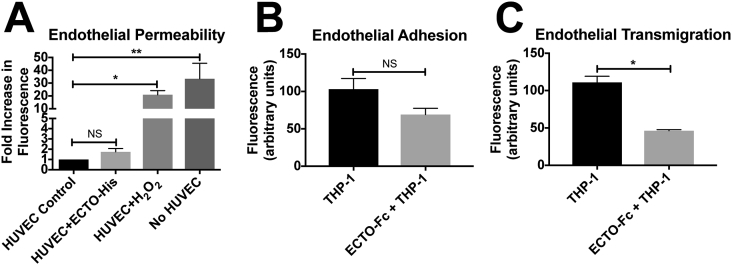
*The soluble ADAM9 ectodomain regulates endothelial monolayer function*. **A.** HUVEC were cultured to confluence on fibronectin coated transwells before incubation with 3 μM ADAM9 ectodomain for 2 h before the addition of FITC-Dextran to measure permeability. HUVEC were also untreated (Control) or treated with 300 μM H_2_O_2_ for 30 min (H_2_O_2_) as a positive control for induction of permeability. Fluorescence measurements (arbitrary units) are from 3 independent experiments. Error bars are ±SEM. * = P < 0.05. ** = P < 0.01. **B.** HUVEC were cultured to confluence on fibronectin coated transwells before incubation with CMFDK green tracker dye labelled THP-1 cells and 3 μM ADAM9 ectodomain for 4 h. THP-1 cells that had migrated into the lower chamber were counted indirectly via measurement of CMFDK fluorescence. Data is the mean of three independent experiments. **C.** Confluent HUVEC in 96 well tissue culture microtire plates coated with fibronectin were incubated with THP-1 CMFDK green tracker dye labelled THP-1 cells and 3 μM ADAM9 ectodomain for 30 min before fixing and measurement of adherent cell number. Data is the mean of three independent experiments. Fluorescence measurements (arbitrary units) are from 3 independent experiments. Error bars are ±SEM. * = P < 0.05. ** = P < 0.01.

As the ADAM9 ectodomain has the ability to self-associate, we hypothesised that the soluble ADAM9 ectodomain could compete with ADAM9-ADAM9 interactions between endothelial cells, thus impairing endothelial monolayer function and possibly increase permeability, as reported for the addition of soluble VE-Cadherin to endothelial monolayers [17]. HUVEC were seeded onto fibronectin-coated transwells and allowed to form a confluent monolayer. Contrary to our expectations, addition of the ADAM9 ectodomain to endothelial monolayers had no significant effect on permeability (Fig. 4A). As cell-cell junctions in endothelial cells can also control leukoycte-endothelial transmigration [18] and the ADAM28 ectodomain can regulate leukocyte-endothelial adhesion or transmigration [10], we speculated the ADAM9 ectodomain might also regulate these processes. THP-1 adhesion to endothelial monolayers was not inhibited by ECTO-Fc significantly, although a decrease in adhesion was detected (Fig. 4B). In contrast, addition of the soluble ECTO-Fc ectodomain significantly inhibited transmigration of THP-1 cells across confluent HUVEC on fibronectin-coated transwells (Fig. 4C).

## 4 Discussion

ADAM9 is expressed in endothelial cells and has been shown to play a role in neovascularisation both in retinal development and in pathologies such as cancer. ADAM9 has been shown to be involved in the ectodomain shedding of endothelial-expressed proteins including Tie-2, Flk-1, VCAM-1, VE-Cadherin and ACE [14], [19]. In this study we have characterised the localisation of ADAM9 in endothelial cells and found it to be localised to cell-cell junctions in confluent monolayers and this overlaps with localisation of VE-cadherin, ZO-1 and γ-catenin. This localisation is dependent on cell-cell contact as ADAM9 is primarily intracellular in subconfluent cells or in cells at the edge of a scratch wound in endothelial monolayers. This localisation is similar to the localisation of ADAM15 in endothelial cells described by Ham et al. [6]. We have also been able to show the ADAM9 ectodomain can self associate using recombinant proteins *in vitro* and that both adjacent cells must express ADAM9 for it to localise to cell-cell junctions. It is not known if this property is shared by ADAM15. ADAM9 has been shown to mediate the interaction between other cells types including monocytes, melanoma cells and fibroblasts [20], indicating this may be a common function for ADAM9. Both ADAM10 and 15 can regulate endothelial permeability via proteolytic and non-proteolytic mechanisms respectively [9], [17], [21]. Although ADAM9 can self-associate via its ectodomain *in vitro,* and this appears to occur in confluent endothelial monolayers, the addition of the recombinant ADAM9 ectodomain to confluent monolayers could not increase endothelial monolayer permeability as has been demonstrated for the soluble ectodomain of VE-Cadherin. Physiologically, ADAM9 can occur as a soluble ectodomain in addition to the type-I transmembrane form [11]. ADAM9 increases 10 fold in polymorphonuclear neutrophils and is produced as a soluble form [12]. This indicates our studies with the ADAM9 ectodomain have a physiological basis. Interestingly, although the ADAM9 ectodomain has been shown to inhibit integrin mediated cell adhesion, it is unable to inhibit monocyte-endothelial adhesion as strongly as transmigration. This is similar to observations by Micocci et al. that showed ADAM9 played a role in MDA-MB-231 endothelial transmigration, but not adhesion [22]. ADAM9 has been shown to interact with a number of integrins, including those containing the β1 subunit [23], [24]. The α6β1 integrin has been shown to be required for neutrophil transmigration, particularly in the later stages of paracellular transmigration [25] and the ADAM9 disintegrin domain can also regulate neutrophil migration and chemotaxis via α9β1 [26]. Monocytes and THP-1 cells also express α6β1 [27], [28]. The disintegrin domain of ADAM9 is able to interact with α6 and α9, but not α5 or α4 integrins [23], [24], suggesting ADAM9 may control α6β1 and/or α9β1 dependent processes during adhesion and transendothelial migration, rather than those dependent on α5β1 or VLA/α4β1 that are known to associate with ADAM17 and 28 [7], [10].

In conclusion we have identified ADAM9 as a component of cell – cell junctions in endothelial cells and have demonstrated that it can self-associate through ecotodomain interactions. Furthermore, although the soluble ADAM9 ectodomain occurs physiologically, it regulates endothelial – monocyte interactions rather than endothelial permeability. These novel findings point to ADAM9 playing an important role in endothelial cell biology that is distinct from the other ADAMs.

## Funding

WRE was funded by the British Heart Foundation Intermediate Research Fellowship FS/03/055/15910 and Cancer Research UK. RS was funded by the Medical Research Council. MH was funded by the Medical Research Council and the Danish Cancer Society. GM was funded by the Medical Research Council and Cancer Research UK.
